# Artificial Intelligence in Edible Mushroom Cultivation, Breeding, and Classification: A Comprehensive Review

**DOI:** 10.3390/jof11110758

**Published:** 2025-10-22

**Authors:** Muharagi Samwel Jacob, Anran Xu, Keqing Qian, Zhengxiang Qi, Xiao Li, Bo Zhang

**Affiliations:** 1 College of Mycology, Jilin Agricultural University, Changchun 130118, China; sammuharagi@gmail.com (M.S.J.); xar0413@outlook.com (A.X.); 15670567112@163.com (K.Q.); qzx7007@126.com (Z.Q.); 2 Engineering Research Center of Edible and Medicinal Fungi, Ministry of Education, Jilin Agricultural University, Changchun 130118, China; 3 Industrial Development Institute for Plants, Animals and Fungi Integration of Biyang County, Zhumadian 463799, China; 4 Hinggan League Institute of Agricultural and Husbandry Sciences, Inner Mongolia Innovation Center of Biological Breeding Technology, Ulan Hot City 137400, China

**Keywords:** artificial intelligence, edible mushrooms, mushroom breeding, mushroom cultivation, species classification

## Abstract

Edible mushrooms have gained global popularity due to their nutritional value, medicinal properties, bioactive compounds and industrial applications. Despite their long-standing roles in ecology, nutrition, and traditional medicine, their additional functions in cultivation, breeding, and classification processes are still in their infancy due to technological constraints. The advent of Artificial Intelligence (AI) technologies has transformed the cultivation process of mushrooms, genetic breeding, and classification methods. However, the analysis of the application of AI in the mushroom production cycle is currently scattered and unorganized. This comprehensive review explores the application of AI technologies in mushroom cultivation, breeding, and classification. Four databases (Scopus, IEEE Xplore, Web of Science, and PubMed) and one search engine (Google Scholar) were used to perform a thorough review of the literature on the utility of AI in various aspects of the mushroom production cycle, including intelligent environmental control, disease detection, yield prediction, germplasm characterization, genotype–phenotype integration, genome editing, gene mining, multi-omics, automatic species identification and grading. In order to fully realize the potential of these edge-cutting AI technologies in transforming mushroom breeding, classification, and cultivation, this review addresses challenges and future perspectives while calling for interdisciplinary approaches and multimodal fusion.

## 1. Introduction

**Figure 1:**
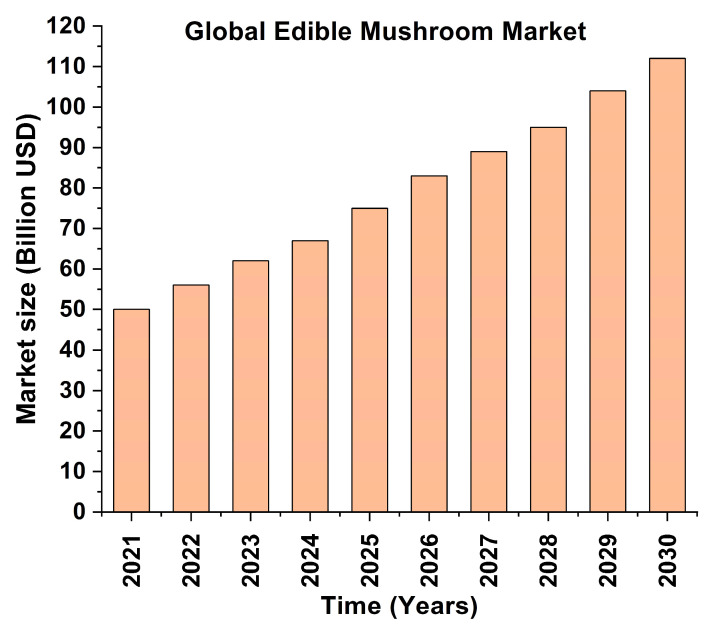
Global market size (billion USD) projected by market reports (market.us) [7].

In recent decades, the mushroom industry has gained prominence as a specialized field in agricultural biotechnology owing to the ecological, nutritional, and pharmacological importance of fungi [1,2,3]. Rich in proteins, dietary fiber, essential amino acids, and a variety of bioactive compounds with immunomodulatory and antioxidant properties, edible fungi are among the best functional food groups with a wide range of ecological and medical applications [4,5,6]. The global edible mushroom market achieved USD 50 billion in value during 2021 and is projected to exceed USD 80 billion by 2030, driven by increased consumer awareness of the health benefits of edible mushrooms, transitioning into vegan diets, and the urge for sustainable food solutions [7,8] (Figure 1). Apart from their nutraceutical significance, mushrooms play crucial ecological roles through their ability to transform agricultural waste into nutritious food products, which benefits the circular bioeconomy [9]. Mushroom species such as *Pleurotus ostreatus* and *Lentinula edodes* have the potential to transform agricultural waste materials, including sawdust, wheat straw and corn husks, to create valuable food products, thus contributing to food security, economic growth and sustainable development [10].

Despite its promising achievements, the mushroom industry is continuously constrained by numerous persistent challenges. Conventional breeding techniques often involve cross-breeding, random mutagenesis, and morphological selection, which are labor-intensive and time-consuming, while producing unpredictably inconsistent results [11]. The cultivation process demands strict control of environmental variables such as temperature, humidity, CO_2_ concentration, and substrate composition, which are typically managed by farmers through traditional methods [12]. Incompetent farm management leads to excessive contamination, poor-quality products, and a decline in yield [13]. Species identification, along with classification for breeding and commercialization purposes, relies heavily on morphological and manual techniques, yet these methods demonstrate a restricted capability to differentiate between morphologically similar species [14] accurately. These challenges are particularly dominant in African and Southeast Asian regions, where indigenous mushroom diversity remains poorly documented due to financial, technological, and taxonomic restrictions that hinder both conservation initiatives and market expansion strategies [15].

AI technologies have recently evolved into powerful tools that address fundamental constraints in agriculture and related fields [16]. The term AI describes how machines replicate human intelligence functions through computer systems, encompassing machine learning (ML), deep learning (DL), and computer vision (CV) [16,17,18]. These technologies have proven their effectiveness in various aspects of crop science, including precision agriculture, weed detection, disease diagnosis, phenotyping, yield prediction, automated harvesting, and quality grading [19]. The application of convolutional neural networks (CNNs), a form of DL, has proven effective in identifying early-stage crop diseases in tomato, wheat and rice with accuracy rates above 95% under controlled laboratory environments [20]. With the use of CV technologies, including satellite imagery and drones, mushroom farms are effectively monitored in real-time, hence decreasing costs of employing manual labor and maximizing production [21]. The successful implementation of these methods proves that equivalent technological advancements could transform mushroom cultivation, breeding processes, and species classification [22].

In mushroom breeding, conventional breeding approaches operate at a slow pace and often rely on morphological traits that might provide inadequate information for selection purposes. The combination of AI predictive modeling with next-generation sequencing (NGS) and phenotyping data enables faster marker-assisted selection, which in turn decreases breeding duration. A recent study by Albayrak et al. [23] on fungal systems showed that ML algorithms, such as Random Forest (RF) and Support Vector Machines (SVMs), achieved better predictions of high-yielding strains and disease resistance traits. Additionally, standardization encounters difficulties attributed to environmental factors and inconsistent cultivation practices [24,25]. AI tools such as reinforcement learning (RL) and Internet of Things (IoT) enable real-time automated monitoring of growing conditions and optimize climate control systems, creating dynamic feedback loops to maintain optimal microenvironments in mushroom farms [26,27]. On the other hand, CNN models aid in the detection of mycelial contamination and substrate colonization status, which enhances timely interventions to decrease spoilage rates [28].

Traditional methods of species classification through microscopy and culture-based techniques are currently experiencing rapid transformation through AI [29]. Recent studies have employed CNNs and SVM to classify edible mushroom species from macro-morphological images with over 90% accuracy [30]. Extensive research combining DNA barcoding and natural language processing (NLP) for taxonomic database mining could enhance classification pipelines, especially in biodiversity-rich areas that lack sufficient research [31]. These advancements optimize both the commercial value chain and conservation efforts through accurate documentation of wild edible species [32].

Numerous studies have explained the application of AI in mushroom studies [33,34,35]. However, a systematic analysis of the application of AI in mushroom cultivation, species identification, and breeding is still lacking. Such analysis will help to bring about new research on how to optimally utilize AI in breeding, cultivation, and species classification. In light of this, our study intends to comprehensively assess and synthesize current research and practical applications of AI across the edible mushroom industry chain focusing on three core domains: (1) AI in mushroom breeding, including genomic prediction through germplasm characterization, phenotype–genotype analysis, multi-omics, gene mining, and genome editing; (2) AI in cultivation practices, focusing on environmental control automation, yield prediction, and disease management; and (3) AI in species identification and classification, with emphasis on image-based recognition, mycelial differentiation, genome-phenotype analysis, and hybrid optimization. Additionally, our study addresses significant challenges and avenues for AI integration in mushroom research. The knowledge gaps revealed in this study provide a basis for effective utilization of AI technologies in multiple aspects of edible mushroom research.

## 2. The Concept of AI

**Figure 2:**
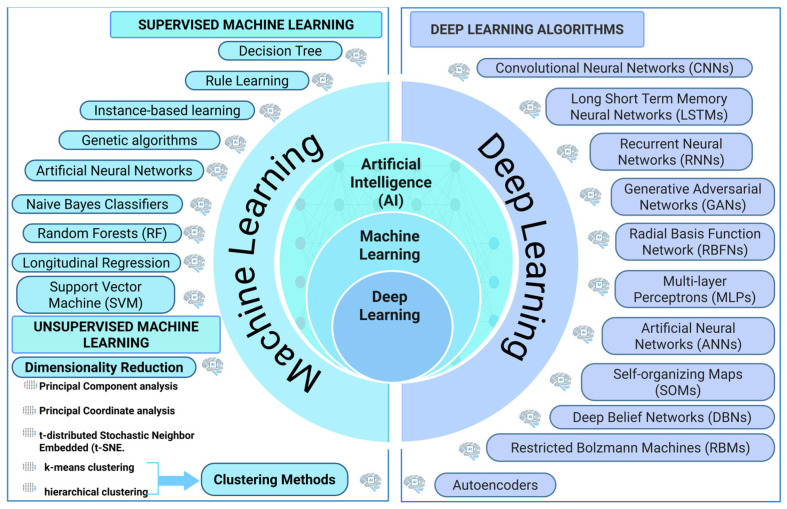
A schematic diagram showing the classification of various AI algorithms.

AI represents a wide computational framework that enables machines to mimic human intellectual capabilities, including learning, reasoning, and problem-solving abilities [36]. AI is a general term that includes ML and DL as its main subfields, which differ in their methodological approaches and algorithmic complexity (Figure 2). ML, a main subset of AI, is categorized into supervised and unsupervised learning approaches [37].

The supervised ML category includes algorithms that use labeled data to train decision trees (DT), SVMs, RF, artificial neural networks (ANNs), and genetic algorithms [38]. Additionally, their capacity to generate predictions and classifications depends on previous input-output data. Unsupervised ML methods employ dimensionality reduction techniques, including Principal Component Analysis (PCA), t-distributed stochastic neighbor embedding (t-SNE), k-means clustering, and hierarchical clustering to analyze unlabeled data and unveil hidden patterns [39]. DL represents an advanced AI system that extends beyond traditional ML methods through its brain-inspired structural and functional principles [40]. The system implements various neural network layers to detect complex patterns in large datasets [18]. Figure 2 presents multiple AI algorithms, including DL architectures such as CNNs for image analysis, Long Short-Term Memory (LSTM) and Recurrent Neural Networks (RNNs) for sequential data, and generative models such as Generative Adversarial Networks (GANs) and Autoencoders. Despite requiring large computational power and extensive datasets, DL models are still capable of delivering superior results in processing nonlinear, high-dimensional data [41].

### 2.1. Advances in AI Techniques and Their Transformative Role in the Edible Mushroom Industry

**Figure 3:**
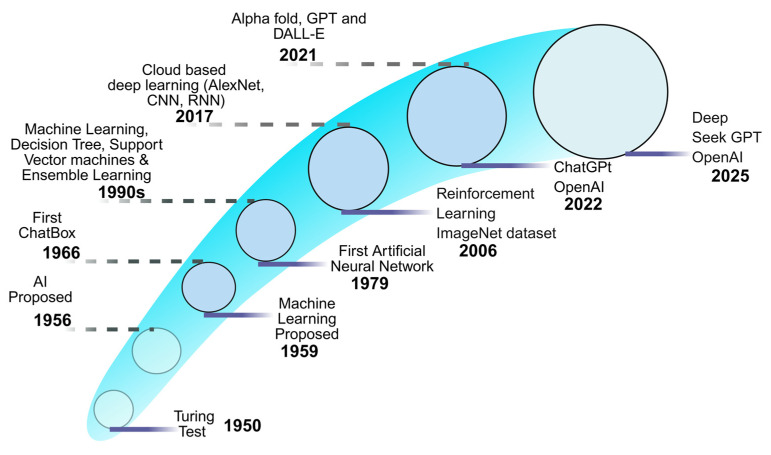
Advances in AI technologies from the 1950s to 2025.

The development of AI started with symbolic reasoning systems from the mid-twentieth century, before it evolved into DL models, which exist in the present (Figure 3). The scientific study of AI began in the 1950s when Alan Turing introduced the Turing test as an intelligence assessment tool for machines [42,43]. John McCarthy and Marvin Minsky invented symbolic AI systems at the 1956 Dartmouth Conference, which introduced formal computational techniques based on logical and rule-based methods [44]. From the 1960s to the 1970s, AI research focused on creating universal problem-solving algorithms. Meanwhile, expert systems such as DENDRAL and MYCIN confirmed AI’s potential in chemistry and medical diagnosis [45]. However, due to insufficient research funds, the system encountered inflexibility and instability, leading to the “AI winter” of the 1980s [46].

In the 1990s, data-driven methods gained popularity due to the development of ML algorithms capable of identifying patterns in empirical data without requiring manual data encoding [47]. DT, SVM, and ensemble methods became prominent by demonstrating improved adaptability and generalizability. The early 2000s brought improvements through the integration of formal RF methods with probabilistic model development [48]. The 2010s brought a resurgence of DL algorithms, which led to fundamental changes in neural networks (Figure 3). Significant advancements in AI were spurred by the development of neural networks, specifically the introduction of CNNs for image classification (AlexNet 2012), and RNNs for sequential data processing [49]. Additionally, the development of autonomous systems and AlphaGo confirmed AI’s capacity to carry out complex tasks [50].

#### Remarkable Developments in AI Between 2020 and 2025

Between 2020 and 2025, AI triggered significant shifts in various sectors through the development of large-scale foundation models such as generative pre-trained transformer (GPT), bidirectional encoder representations from transformers (BERT), and DALL·E, which coordinate multimodal data while exhibiting human-level abilities in the speech, vision, and text domains [51]. These developments, supported by cloud computing, big data, and advanced hardware (Graphic Processing Unit (GPU), Tensor Processing Unit (TPUs), have enabled AI to permeate diverse sectors, including agriculture, healthcare, finance, and scientific research, making AI an indispensable tool in modern innovation ecosystems [52,53].

In 2020, deep learning emerged as the dominant paradigm, with convolutional neural networks (CNNs) and recurrent neural networks (RNNs) achieving an outstanding performance in image recognition, disease detection, and environmental data analysis [54]. These models significantly enhanced precision in visual classification tasks, forming the technological foundation for intelligent mushroom diagnostics and automated monitoring systems [55].

By 2021, transformer-based architectures such as BERT and Vision Transformers (ViT) gained prominence for their superior capability to process sequential and visual data simultaneously [56]. The introduction of self-regulating mechanisms improved both interpretability and scalability, enabling highly accurate classification of complex fungal morphologies [57]. In 2022, the emergence of generative adversarial networks (GANs) and self-supervised learning methods substantially improved data efficiency and model robustness [58], addressing the persistent challenge of limited labeled datasets in biological research [59,60].

The year 2023 marked the integration of reinforcement learning (RL), YOLOv5, and hybrid AI frameworks with Internet of Things (IoT) technologies [61]. These systems autonomously optimize environmental parameters such as humidity, temperature, and CO_2_ concentration. Additionally, the invention of AI-powered robotic arms has revolutionized mushroom harvesting [62]. AI-powered robotic arms use advanced motion control and specially designed grippers to harvest, trim, and pack mushrooms gently [63]. These robots operate tirelessly within mushroom farms, significantly reducing labor costs and operational delays, thereby enhancing productivity and sustainability in mushroom cultivation [64].

By 2024, the rise in large multimodal models, including GPT-4 and Contrastive Language Image Pre-training (CLIP), facilitated the integration of image, text, and sensor data, revolutionizing predictive analytics and intelligent decision-making in agricultural systems [65]. Moreover, sophisticated deep learning models, including ResNet-50 and MobileNet-V2, were applied to digital imaging to predict the shelf life of mushrooms. This breakthrough improved quality control during post-harvest handling and distribution [66].

In 2025, explainable AI (XAI) and edge AI technologies became mainstream, merging interpretability with real-time, on-site analytics [67]. These innovations enabled transparent, efficient diagnostic and management systems, marking a pivotal transition toward autonomous, data-driven, and sustainable mushroom production ecosystems [68,69]. As AI continues to revolutionize mushroom production systems, its application in the core areas of breeding, cultivation, and species classification warrants deeper exploration [47].

## 3. Application of AI in Mushroom Cultivation

The transition from manual to automated and large-scale edible mushroom cultivation systems depends heavily on AI to provide smart farming solutions. The main driver of this transformation involves integrating AI with environmental sensing technologies, vision-based monitoring and predictive modeling systems [70]. These implemented systems enhance operational efficiency while delivering consistent yields at minimal production costs and reducing the risk of disease outbreaks [13,71].

### 3.1. Intelligent Environmental Control

**Figure 4:**
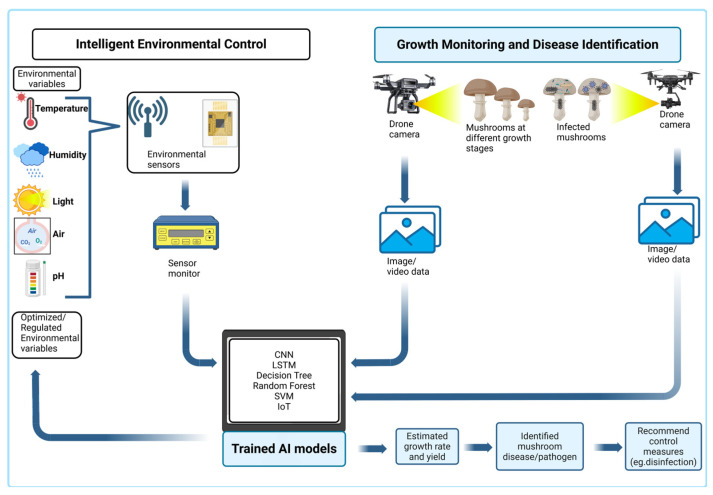
Application of AI in cultivation in edible mushrooms.

**Figure 5:**
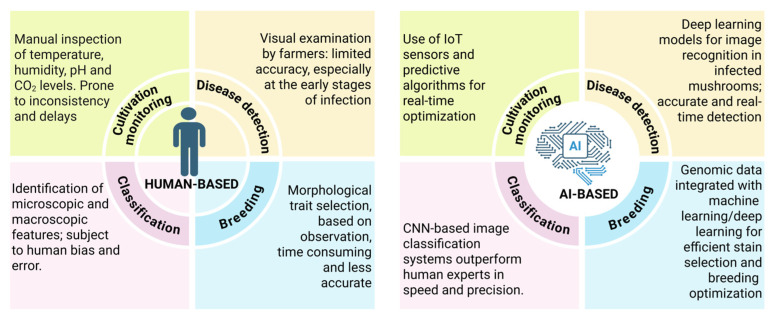
A comparative presentation of human-based and AI-assisted task completion during mushroom identification, cultivation, and breeding.

The successful cultivation of edible mushrooms depends on optimal environmental conditions that are essential for mycelial growth, fruit body development, and yield increase [72] (Figure 4). An efficient control of temperature, relative humidity, light intensity, carbon dioxide (CO_2_) levels, and substrate moisture promotes the formation of healthier fruiting bodies [73]. Traditional management systems employ manual or semi-automated methods, which lack adaptability and precise control over internal or external environmental changes (Figure 5). AI systems integrated with IoT sensors provide a practical solution to this challenge [74].

The cultivation chamber or greenhouse integrated with an IoT system controls environmental parameters through continuous real-time data collection from multiple sensors [75]. Environmental data from various parameters, including substrate temperature, CO_2_ concentration, humidity, and illumination levels, are closely monitored by sensors and transmitted to a centralized processing unit (Figure 4). AI algorithms utilize LSTM networks, a type of RNN, to analyze historical and real-time data for environmental trends [76,77]. LSTM models demonstrate high accuracy in predicting diurnal temperature and humidity fluctuations, allowing operators to induce advanced modifications to ventilation, humidification, and heating systems [78]. This observation aligns with a study carried out by Nguyen et al. [79], in which sensor data models were employed to control temperature, humidity, and air circulation in a *Pleurotus eryngii* greenhouse farm. Implementation of this model reduced air conditioner energy consumption by more than 20% while enhancing ideal growth parameters and yield consistency [80]. Similar adaptive control models based on fuzzy logic and RL were employed in Chinese and Japanese mushroom growing facilities to optimize resource allocation, maintain consistent microclimates, and extend post-harvest shelf life [70,81]. The integration of AI with IoT technology enables precise environmental management while contributing to sustainable mushroom production. These systems reduce the environmental impact of indoor farming by conserving energy and water resources, thus supporting the objectives of low-carbon agriculture and smart food systems [82].

### 3.2. Growth Monitoring and Disease Identification

To produce high-quality mushroom products and maximize profit, accurate monitoring of growth stages and disease detection is essential [83]. Manual examinations have been used for a couple of years; however, they are time-consuming and less accurate (Figure 5). Currently, high-resolution imaging technologies are used to collect daily visual data and monitor mushroom farms [28]. The data collected are then analyzed using DL models, such as ResNet (Residual Networks) and YOLO (You Only Look Once). Through these models, potential morphological characteristics such as cap diameter, stem length, texture, and color are thoroughly examined [84]. These characteristics serve as indicators for both growth rate and health condition. A study conducted by Ahmed & Kou [85] achieved more than 94% accuracy in detecting the maturity level of *Lentinula edodes* and classifying diseases through ResNet50-based CNN models, which enabled them to plan harvest times and determine workforce needs (Table 1).

It was further discovered that CV technologies such as drones and other unmanned aerial vehicles (UAVs) are capable of maintaining optimum growth conditions, early disease detection, and estimation of growth rate [86]. The commercial mushroom industry is currently facing two major economic threats: brown spot disease, caused by *Pseudomonas tolaasii* and green mold contamination by *Trichoderma* spp. [86]. The initial signs of these diseases appear as tiny discolorations or growth irregularities that are hardly detectable by the naked eye [83,86]. Through CV technology, AI-enhanced image recognition tools can identify minor visual indicators to locate specific symptomatic regions and ensure accurate identification of diseases (Figure 4). Thus, utilizing YOLOv5 for early detection of mold infection in mushroom farms can facilitate immediate diagnosis and undertaking of appropriate preventive measures [71].

Furthermore, the integration of time-series image data with environmental sensor inputs promotes the development of predictive models that establish correlations between growth anomalies and particular environmental determinants [87]. This allows the system to detect and analyze the root cause, leading to corrective actions such as substrate sterilization, moisture adjustment, and fine-tuning of the light spectrum (Table 1).

Variability in performance between different AI models under varying cultivation environments demonstrates that model appropriateness depends on data availability and task complexity (Table 1). DL models, including CNN, LSTM, and hybrid architectures (LSTM-RL), achieve superior precision (>90%) when performing pattern recognition and time-series analysis tasks such as mycelial growth monitoring, environmental control, and harvest timing (Table 1). These models attain their highest performance when supplied with large annotated datasets and sufficient computational power for real-time data analysis [88]. The structured tasks of yield prediction, spawn quality classification, and substrate optimization demonstrate reliable performance (80–90%) from classical ML algorithms, including RF, SVM, and adaptive boosting (AdaBoost) when working with moderate tabular data [77]. The deployment speed of DT and k-nearest neighbor (k-NN) models makes them suitable for quick prototyping and resource-limited environments, although they achieve lower accuracy levels [16,80]. The emerging field of tiny machine learning (TinyML) and IoT-integrated systems provides cultivation environments with real-time low-power monitoring capabilities through edge computing [89]. Generally, hybrid and DL approaches offer the highest precision yet require a large amount of data and tuning compared to conventional models, which provide interpretability and easy integration.

AI applications in mushroom cultivation face multiple challenges despite achieving high performance levels. Environmental changes associated with model drift lead to decreased long-term accuracy of the system [90]. DL models require extensive large datasets, yet biological annotation data remains insufficiently available [18]. Intelligent pre-processing and validation techniques are necessary to ensure the generalizability and reliability of the model, as inconsistent labeling and data noise from sensor variability can also lead to inefficiency in model training [91].

**Table 1:** Application of AI models at various stages of edible mushroom cultivation.

Cultivation Parameter	AI Algorithm/Model	Application	Performance (%)	Data Requirement	Reference
Spawn quality assessment	SVM, RF	Classification of high-quality spawn	87–100	Microscopic images + mycelial growth data	[79]
Substrate optimization	ANN, DT	Predicting yield from substrate composition	90–92	Physicochemical substrate parameters	[92]
Growth monitoring	CV, CNN	Monitoring mycelia colonization in bags	93.5	RGB images under controlled lighting	[28]
Fruiting body detection	YOLOv5, CNN	Real-time detection of fruiting body development	94.8	Annotated image datasets (>2000 images)	[35]
Harvest time prediction	LSTM	Forecasting optimal harvesting day	88.6	Historical growth + Environmental data	[93]
Pest and disease detection	CNN, ResNer50, TL	Early detection of larvae, mites, nematodes, fungal and bacterial infections	95.2	High resolution fruit body images	[88]
Climate control optimization	IoT, TinyML, RL	Adaptive control of humidity (moisture), CO_2_, and temperature	89.3	IoT sensor streams + feedback loops	[78,94,95]
Yield forecasting	Gradient Boost, AdaBoost	Multi-variable yield prediction model	91.7	Substrate, strain, environment, and time	[96]
Energy efficiency management	TinyML	On-device inference for light/ventilation optimization	85–88	Real-time sensor data from IoT devices	[89]
Contamination detection	Autoencoders, PCA	Detection of contamination during spawn production	86.5	Spectral+ image data from production units	[81]
Lighting condition adjustment	Fuzzy logic +CNN	Intelligent lighting control to stimulate production	88.4	Spectral sensor +image sequence	[70]
Multi-stage process modeling	Ensemble models (RF + XGBoost)	Integrative analysis of multiple cultivation variables	93.1	Longitudinal multi-modal data	[97]

## 4. Application of AI in Mushroom Breeding

### 4.1. AI-Enabled Germplasm Characterization in Edible Mushrooms

**Figure 6:**
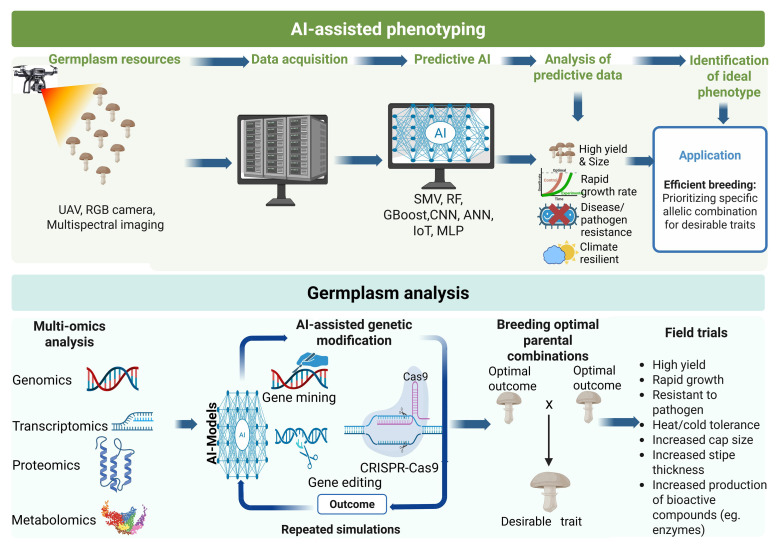
Application of AI in various aspects of edible mushroom breeding.

The characterization and utilization of genetic resources (germplasm) are foundational to edible mushroom breeding. Traditionally, morphological and biochemical traits have been used to evaluate germplasm in various mushroom species, namely *Agaricus bisporus*, *Pleurotus ostreatus*, and *Ganoderma lucidum* [98]. However, these methods are labor-intensive, prone to error, and often limited in their ability to reveal hidden genetic potential. AI now plays a central role in transforming germplasm characterization by integrating and analyzing genomic, phenotypic, and environmental data [99,100] (Figure 6).

ML algorithms, such as unsupervised clustering (K-means, hierarchical clustering), have been applied to classify diverse mushroom strains based on DNA sequence data, metabolite profiles, and environmental adaptability [99]. For example, Zhu et al. (2025) utilized PCA combined with SVM models to cluster wild *Pleurotus* isolates into distinct phenotypic groups based on their enzymatic activity and substrate degradation potential. This facilitated the identification of promising strains for bioconversion and commercial production [100].

AI-based feature selection methods such as recursive feature elimination (RFE) have also been employed to isolate genetic markers, including simple sequence repeats (SSRs) and single-nucleotide polymorphisms (SNPs) associated with high-yield and stress-resilient traits [101]. When integrated with genome-wide association studies (GWAS), AI enables breeders to uncover rare alleles or combinations that contribute to performance under specific substrates or environmental conditions [102]. Moreover, AI supports digital genebank management by creating relational databases that autonomously update trait and performance data points. Such systems not only improve traceability and access to elite strains but also reduce redundancy in breeding programs [103]. Thus, AI is crucial for harnessing the potential of mushroom genetic resources and accelerating their targeted deployment in breeding pipelines [104].

### 4.2. Application of AI in Digital Phenotyping for Edible Mushrooms

Phenotyping observable traits such as cap diameter, mycelial density, fruiting time, and contamination levels is critical in edible mushroom breeding [105]. Traditional phenotyping has resulted in several shortcomings in edible mushroom breeding, associated with the inability to handle multiple data. AI has revolutionized digital phenotyping in mushroom research by enabling efficient, precise, and automated assessment of morphological and physiological traits using sensor technologies and image-based analytics [106].

CV and CNNs are currently extensively used to extract phenotypic features from digital images of mushrooms grown under varying substrates and environmental conditions. For example, Huamao et al. [106] employed CNNs to classify *Pleurotus geesteranus* phenotypes with accuracy of over 97% based on cap morphology, color, and gill symmetry. These models allow breeders to detect subtle phenotypic differences that would be difficult to quantify manually, enhancing selection accuracy and speed. In addition, AI-powered IoT systems integrate environmental sensors (temperature, CO_2_, humidity) with phenotyping algorithms to dynamically adjust cultivation conditions and predict growth outcomes. This real-time phenotyping feedback loop is critical in systems such as vertical farming units and closed-environment mushroom houses [107].

Furthermore, phenomics platforms incorporating hyperspectral imaging and thermal cameras, combined with ML algorithms, are being tested to monitor early signs of disease or physiological stress [103]. Such systems significantly reduce production losses and enhance the accuracy of experimental breeding trials. Therefore, AI-driven digital phenotyping bridges the precision gap between environmental management and phenotype selection, making it indispensable for efficient mushroom breeding programs [106].

### 4.3. Integrating AI with Multi-Omics Data in Edible Mushroom Breeding

The integration of multi-omic datasets (genomics, transcriptomics, proteomics, and metabolomics) offers a systems-level understanding of the biological processes in edible mushrooms [108,109,110] (Figure 6). However, managing and interpreting these high-dimensional, multi-source datasets is a major challenge. The advent of AI, particularly DL and ensemble ML models, has increased efficiency in decoding complex biological data that underpin important agronomic traits in mushrooms, such as yield, flavor profile, shelf-life, and stress tolerance [111].

In recent studies, AI frameworks such as deep neural networks (DNNs) and autoencoders have been employed to integrate and reduce dimensionality in mushroom omics data [112]. Schiphof et al. [113] used multilayer perceptron (MLP) networks to correlate transcriptomic data from *Pleurotus ostreatus* under oxidative stress with metabolite accumulation, thereby enabling the identification of biomarkers linked to antioxidant capacity. Furthermore, RF and gradient boosting models have been used to predict fruiting body formation by integrating DNA methylation patterns (epigenomics) with growth-stage transcriptomics [114]. These models assist in revealing regulatory mechanisms and gene expression thresholds required for optimal sporophore development and enhanced nutraceutical properties.

Integrative omics, powered by AI, also facilitates the reconstruction of metabolic pathways for species like *Ganoderma lucidum*, where the production of bioactive compounds is of commercial interest [115]. AI-based clustering and pathway prediction algorithms (Pathway tools with AI-based gene function prediction) assist in identifying candidate genes for metabolic engineering [116]. Overall, AI-enabled multi-omic integration transforms mushroom breeding from phenotype-driven to systems-guided, enhancing predictive power and enabling the targeted design of high-performance cultivars [117,118].

### 4.4. AI-Driven Genotype–Phenotype Synergy for Efficient Edible Mushroom Breeding

A major bottleneck in edible mushroom breeding lies in linking genetic variation (genotype) with observable characteristics (phenotype) [119]. This genotype–phenotype gap is especially pronounced in fungi due to their complex life cycles, environmental responsiveness, and epigenetic plasticity. AI offers powerful tools to bridge this gap by modeling nonlinear relationships between genes, regulatory elements, and morphological or biochemical traits [112].

AI approaches such as SVM, DL, and Bayesian networks have been increasingly applied in this domain. In shiitake mushrooms (*Lentinula edodes*), for example, Xu et al. [107] used SVMs to predict cap size and firmness based on SNPs and transcriptome profiles, achieving over 85% accuracy in cross-validation. These models help breeders prioritize which genotypes are likely to produce commercially valuable phenotypes before field trials [107].

CNNs combined with genomic feature extraction are also being used to map fruiting efficiency, mycelial colonization speed, and bioactive compound expression in *Ganoderma* and *Pleurotus* species [120]. Importantly, AI models can integrate environmental variables such as humidity and substrate composition into genotype–phenotype prediction, thereby accounting for gene-environment interactions (G×E) [109]. Reinforcement learning and probabilistic models are further applied to refine these predictions in dynamic cultivation environments, where trait expression varies across growing cycles [121]. By quantifying the influence of genotypes on phenotypes with increasing accuracy, AI bridges knowledge gaps that have limited strain improvement for decades [109,121]. This shift allows mushroom breeders to pursue precision breeding strategies informed by data-driven predictive biology.

### 4.5. AI-Driven Gene Mining for Edible Mushroom Breeding

Gene mining in edible mushroom species focuses on identifying genetic loci that control important traits such as yield, stress resistance, substrate degradation efficiency, flavor, and medicinal compound biosynthesis [122]. Traditional gene discovery methods are labor-intensive and limited by the complexity of fungal genomes. AI enhances this process by rapidly analyzing large-scale genomic and transcriptomic data to predict gene function, prioritize candidate loci, and infer regulatory networks [123,124].

ML algorithms such as RF, SVM, and Naive Bayes classifiers have been employed to predict gene–trait associations. For instance, Rivera et al. [125] applied RF models to predict ligninolytic enzyme gene families in *Pleurotus ostreatus*, identifying novel laccase and peroxidase genes involved in efficient lignin degradation, an essential trait for industrial composting and substrate recycling. DL models such as LSTM networks and autoencoders have also been used to extract features from time series data for gene expression in *Pleurotus ostreatus*, helping to uncover genes linked to primordia initiation and fruiting body development under stress conditions [126]. These tools surpass conventional statistical methods by capturing nonlinear interactions and context-specific gene expression dynamics. Furthermore, AI-enhanced sequence alignment and annotation tools (DeepGOPlus, PhyloAI) integrate evolutionary and structural data to predict functional domains and conserved motifs in newly sequenced mushroom genomes [127], particularly in non-model genera like *Grifola* and *Hericium* [128]. By accelerating the discovery of functional genes, AI empowers targeted breeding, genome editing, and metabolic engineering of edible mushrooms, thereby supporting the development of strains tailored for nutrition, medicine, and environmental sustainability [129].

### 4.6. Application of AI in Genome Editing in Edible Mushrooms

Traditional mushroom breeding methods such as random mutagenesis, strain hybridization, and transgenic approaches have contributed significantly to improving yield, flavor, shelf life, and stress tolerance in edible mushrooms [130]. However, these conventional techniques are constrained by lengthy breeding cycles, high unpredictability, incomplete gene knockouts, and labor-intensive screening [131,132]. With the development of precise genome sequencing and molecular biology tools, precision breeding in fungi has gained momentum. Among these innovations, CRISPR/Cas9 and related gene editing platforms (Figure 6) have emerged as transformative technologies for enhancing mushroom traits, including disease resistance, improved lignocellulose degradation, and enriched nutraceutical content [133,134].

The discovery of advanced gene editing techniques, particularly the site-directed nucleases (SDNs) encompassing zinc-finger nucleases (ZFNs), transcription activator-like effector nucleases (TALENs), and CRISPR-based systems, enables targeted editing of mushroom genomes, overcoming the inefficiencies of random mutagenesis [133]. Recently, CRISPR/Cas9 has been successfully applied in *Pleurotus ostreatus* to improve fruiting body formation and β-glucan synthesis efficiency [120]. AI significantly enhances genome editing by enabling the development of compact, highly specific nucleases, facilitating functional annotation, and predicting protein structure. The genome-editing components for edible fungi can be optimized by using tools like AlphaFold2, which predict nuclease and effector protein conformations with nearly experimental accuracy [135].

Furthermore, AI-driven large language models (LLMs) trained on fungal genomic and CRISPR datasets can design novel editors tailored for complex mushroom genomes [136]. These methods improve editing efficiency, reduce off-target effects, and pave the way for synthetic biology applications aimed at engineering ideal mushroom strains with enhanced nutritional, medicinal, and environmental attributes. Combining AI with CRISPR/Cas and protein engineering thus offers a data-driven, precise, and scalable route toward next-generation edible mushroom breeding programs [137,138].

## 5. Application of AI in Edible Mushroom Classification

### 5.1. Automatic Identification of Species

Distinct morphological traits of edible fungi, such as stipe length, spore pigmentation, gill arrangement, and cap shape, offer a wealth of information for species identification [139]. CNNs, a type of DL, have demonstrated potential utility in automatic identification and classification of macro-fungi [140]. These networks are capable of learning hierarchical representations of visual features from raw pixel data, enabling them to distinguish between subtle morphological traits that define species boundaries [49].

Recently, a study on CNN models trained using annotated images of wild mushrooms demonstrated high accuracy in distinguishing a wide variety of mushroom species (Table 2). After training with 2500 images, the CNN model achieved over 92% accuracy in test trials [32]. These models analyze cap shape, gill spacing, color gradients, and surface textures through region-based image segmentation to identify relevant morphological structures [23,141].

To resolve challenges associated with a small dataset, TL has proven effective [142]. Pre-trained TL models such as ResNet, visual geometry group (VGG16), or InceptionV3 are currently employed to enhance image recognition and processing based on large datasets like ImageNet [49]. The model demonstrates its excellence through reduced training times and improved precision for identification of new species [143].

Furthermore, the discovery of mobile-based AI applications for on-site mushroom identification represents a significant advancement in mushroom taxonomy [144]. Real-time analysis of mushroom images through CNNs integrated with TL occurs after digital camera devices capture on-site images [79,145]. Apart from merely distinguishing edible and poisonous species, these applications also supply information regarding toxicity, species locations, and ecological research data. The AI-assisted mobile app achieved 95% accuracy in field tests when it analyzed cap and gill morphology to distinguish edible species (*Boletus edulis, Cantharellus cibarius*) from non-edible species (*Amanita muscaria, Galerina marginata*) [146].

Various AI models used for edible mushroom identification and classification demonstrate direct relationships between algorithm complexity, classification accuracy, and data type [142]. Deep learning architectures such as hybrid MobileNetV2–VGG16 convolutional neural networks (CNNs) exhibit superior performance when trained on high-resolution image data. Their combined structure leverages the lightweight efficiency of MobileNetV2 and the powerful feature extraction of VGG16, achieving accuracies above 97% in distinguishing morphologically similar species, including *Pleurotus ostreatus*, *Pleurotus eryngii*, *Lentinula edodes*, *Ganoderma lucidum*, and *Volvariella volvacea* [147]. Nonetheless, the reliance on limited and homogeneous image datasets often results in overfitting and reduced adaptability under diverse environmental or lighting conditions. Employing data augmentation, transfer learning, and large-scale fungal image repositories may improve efficiency and generalization in cultivation systems.

In contrast, classical algorithms such as Support Vector Machines (SVM) perform effectively with small, well-organized datasets, particularly for morphological classification, achieving 76% accuracy for *Pleurotus ostreatus* (Table 2). Despite their simplicity and interpretability, their efficiency declines when applied to complex, unstructured visual data. Integrating CNN-based feature extraction before SVM classification enhances discriminative capacity, bridging the gap between traditional and modern approaches [103,148].

YOLOv5 and Mamba YOLO demonstrate exceptional capability for real-time mushroom detection and sorting, advanced object detection algorithms [149]. These models have achieved over 90% accuracy in detecting multiple species under variable environmental conditions, offering fast localization and identification that are essential for automated harvesting and grading [149]. However, their dependence on large, annotated datasets and vulnerability to environmental variations necessitate the adoption of semi-supervised and adaptive domain training strategies to maintain consistent accuracy [35,126].

Ensemble learning approaches such as AdaBoost have also shown promise, achieving over 95% accuracy in structured datasets by combining multiple weak classifiers to form a robust predictive model (Table 2). While efficient in handling class imbalance and noisy data, AdaBoost remains sensitive to mislabeling and is unsuitable for raw image inputs. Its performance can be improved by integrating it with CNN-derived feature representations, particularly for image-based classification tasks [150].

Advanced AI architectures such as ResNet and DenseNet201 introduce residual and dense connectivity mechanisms that address diminishing gradient problems while enabling the capture of complex morphological features such as pileus texture, lamellae patterns, and stipe structure [143,151]. These models attain accuracy levels of approximately 96–97% across diverse edible and medicinal species, including *Boletus edulis* and *Ganoderma* spp. [151]. However, their computational demands and dependence on large, well-balanced datasets limit their applicability in remote settings. Lightweight optimization techniques such as pruning and quantization, combined with transfer learning, can enhance their practical utility in mushroom industries [151].

Due to their ability to model complex nonlinear relationships, DNNs offer outstanding outcomes in quality assessment and spawn evaluation, achieving accuracies close to 99% [152] (Table 2). Despite their effectiveness, their opaque internal structure (black-box nature) hinders interpretability, raising significant concerns in food quality assurance contexts [152]. Integrating explainable AI (XAI) techniques such as Gradient-weighted Class Activation Mapping (Grad-CAM) can mitigate this restriction and provide visual explanations for model predictions [153,154].

Furthermore, advancements in AI led to the development of sophisticated tools such as EfficientNetB7, EfficientNetV2 and AlexNet-RCNN hybrid models, which systematically balance network depth, width, and resolution [155,156]. These architectures consistently achieved 80–98% accuracy in distinguishing edible species such as *Agaricus arvensis* from poisonous ones, including *Amanita muscaria, Aleuria aurantia*, and *Amanita pantherina* [155,156]. Despite being computationally intensive, their scalability makes them ideal for quality monitoring and digital traceability in macro-fungi industries [157].

Hybrid DT, particularly SVM (DT + SVM) models, which combine the interpretability of tree-based classifiers with the precision of SVMs, have achieved the highest classification accuracies in small-scale datasets [158]. However, such performance often reflects overfitting or dataset bias, underscoring the need for multiple cross-validation and independent data testing to ensure generalization [158].

Additionally, hyperspectral imaging (HI) and near-infrared spectroscopy (NIS) provide molecular-level biochemical and structural details that surpass visual inspection for species differentiation [159]. These approaches excel at identifying small compositional variations between closely related taxa. Both methods show efficiency but experience difficulties when operating in changing environmental conditions and limited data availability [159]. The solution to these challenges exists in integrating AI into molecular taxonomy [128]. The application of DL models to genomic, transcriptomic or metabolomic data enables the precise identification of species [119]. AI systems integrated in classification processes aid in solving morphological and chemical similarity issues by achieving exact identification of cryptic and novel taxa [29]. The advancement of reliable and scalable mushroom species identification and classification requires a multi-modal approach that merges image, spectral, and molecular data under AI frameworks [148].

**Table 2:** Application of different AI models in edible mushroom identification and classification.

AI Algorithm/Model	Application	Data Requirement	Performance Accuracy (%)	Mushroom Species	Reference
MobileNetv2 + VGG-16	Identification of commonly cultivated, edible, and medicinal mushrooms	A dataset comprising 600 image samples of six medicinal mushrooms	97.3 and 72.6 respectively	Oyster (*Pleusrotus ostreatus*), Reishi (*Ganoderma lucidum*), Shiitake (*Lentinula edodes*) Lion’s Mane (*Hericium erinaceus*), Shimeji (*Hypsizygus tessulatus*)*,* and Volva (*Volvariella volvacea*)	[147]
SVM	Morphological feature-based classification	Numerical features from shape or texture	76.6	*Pleurotus ostreatus*	[148]
YOLOv5, Mamba YOLO	Edible mushroom detection and classification in scenes with vertical stick placement	Annotated large resolution video or image datasets	Over 90	*Pleurotus* spp., Shiitake (*Lentinula edodes)*	[149,160]
Adaboost	Classification of edible and inedible mushrooms	8124 data samples from the mushroom repository dataset for the purpose of classification	95.35 in a 10-fold cross validation	*Agaricus compestris*	[150]
ResNet + DenseNet201	Identification of most popular and farmed mushroom species	Image dataset containing 500 basic photographs of various mushrooms	96.72	*Boletus edulis, Pleurotus* spp. (Golden Oyster, Milky Oyster, and Pink Oyster mushroom), *Auricularia* (Ear Mushroom), *Ganoderma* mushrooms (Reishi).	[143,151]
DNN classifiers	Quality classification of mushroom spawn	Digital images of mushroom spawn	98.8 in a 4-fold cross validation	Oyster mushroom (*Pleurotus ostreatus)*	[152]
ResNet50	Edible and poisonous mushroom classification	Data containing various images of mushrooms with labels indicating whether they are edible or poisonous	highest prediction value of 95.64	Wild Boletes (*Boletus* spp.) mushrooms	[49,161]
EfficientNetB7	Classification of edible from inedible mushroom species	676 image data samples from several mushroom species	Over 80	*Agaricus, Amanita, Boletus, Cortinarius, Entoloma, Hygrocybe, Lactarius, Russula, and Suillus*	[156]
CNN +EfficientNetV2	Dimensionality reduction and species classification	6714 mushroom images datasets of complex features	97	Edible; *Agaricus arvensis*, *Agaricus augustus*, *Cantherallus cibarius* Poisonous and inedible; *Aleuria aurantia*, *Amanita muscaria*, *Amanita pantherina*, *Gaestrum michelinum*	[155]
AlexNet (CNN + R-CNN)	Classification of poisonous and edible mushrooms	Images of edible and poisonous mushrooms	98.5 and 95.5 respectively	Edible: *Amanita citrina*, *Russula delica*, *Phaeogyroporus portentosus*, Poisonous: *Inocybe rimosa* and *Amanita phalloides*	[157]
DT + SVM	Edible mushroom identification based on qualitative data	Datasets containing 8124 samples of *Agaricus* and *Lepiota*	100	*Agaricus* spp. and *Lepiota* spp.	[158]

### 5.2. Application of AI in Quality Grading

Quality grading is crucial for commercial mushroom production and supply chains due to its effect on market value and consumer behavior [151]. The quality indicators for mushrooms include cap diameter, texture, uniformity, color, maturity level, bruises, and discoloration, which require accurate examination. Manual inspection methods involve a substantial labor force while generating inconsistent grading outcomes (Figure 5). The combination of AI-based CV systems with multispectral imaging (MSI) and ML algorithms is widely employed to automate these processes with high precision [21,162].

Mushroom tissue analysis of tiny chemical and structural differences becomes feasible through multispectral imaging, which gathers data across wavelengths beyond the visible light spectrum [163]. These systems use PCA to reduce dimensions and SVM for classification, enabling them to perform quality grade sorting of mushrooms [145,164]. In the study by Mollazade [165], an automated grading line for *Agaricus bisporus* used PCA to analyze multispectral images, which identified key morphological features related to cap whiteness, uniformity, and size. The SVM classifier obtained features from the system, which was trained with labeled quality grades (Grade A, B, C). The system achieved a classification accuracy higher than 90% by processing 300 specimens per minute, thus proving its exceptional efficiency [165].

AI systems combine near-infrared imaging with CNNs to detect early spoilage signs and assess traditional grading parameters [147]. Through proactive sorting prior to packaging, the system enhances early identification of low-quality produce, reducing post-harvest losses and extending shelf life [147]. Another creative strategy is the combination of robotic arms and AI-based visual grading systems. The system classifies mushrooms into different packaging categories based on their quality under automated sorting processes [166]. By continuously learning from quality assurance feedback, AI models improve their performance through creation of automated and optimized production systems [167]. The implementation of AI-powered classification and quality grading systems has revolutionized both traditional mushroom taxonomy and commercial inspection procedures [168]. The combination of advanced imaging technologies with DL models enhances the capacity of these systems to deliver sustainable solutions by ensuring safety, increased productivity, and consumer preference for edible mushroom products [167,168].

## 6. Challenges and Future Perspectives

### 6.1. Current Challenges

#### 6.1.1. Availability and Quality of Data

Most researchers in mycology face challenges in utilizing AI for mushroom research; among these are the lack of an integrated, high-quality, and standardized dataset. AI algorithms, especially ML, rely on datasets to achieve accurate predictions. Some challenges associated with mushroom research data are based on their sparse, fragmented, or inconsistent nature. For instance, mushroom data related to growth conditions, genetic variation, disease patterns, and ecological interactions are not systematically collected or standardized [169]. Furthermore, most mushroom data are collected based on individual research projects, which complicates the possibility of assembling large and representative datasets required to train AI models [170]. These data challenges hinder the training of more efficient AI models and, to some extent, impede the reproducibility and scalability of research output. Essentially, the non-standardized dataset will cause models trained on local or institutional data to largely fail when working with different strains or under varying environmental conditions [170].

#### 6.1.2. Ethical and Environmental Considerations

Progress has been made in mushroom research and agriculture, underscoring the need to consider the ethical and environmental implications of AI. AI could, therefore, lead to the increased use of pesticides or artificial growth enhancers in agriculture and mushroom farming, which would be detrimental to the environment [171]. The automation of mushroom farming procedures will likely decrease the number of farmworkers, raising ethical concerns about the future of these workers. The primary challenge lies in making the adoption of AI both sustainable and responsible, while addressing its ethical implications [172].

#### 6.1.3. Data Privacy and Intellectual Property (IP)

The functionality of AI in edible mushroom research relies on the collection and sharing of data. Data privacy and IP considerations are significant in the course of mushroom studies. Some proprietary data, for example, genetic sequences or cultivation data, may deter researchers from availing such proprietary data due to the fear of the misuse or outright theft of intellectual property [160].

Mistrust among parties and an anti-sharing data attitude will slow down the development of AI models, thereby inhibiting partnerships. Proper data and IP management within ethical mushroom research lines will enable a broader adoption of AI [165]. Despite the great promise, mushroom research using AI has some unresolved challenges, including data availability, multidisciplinary collaboration, and cost-related issues [173,174]. Mushroom ecology is another layer of complexity that prevents proper alignment of AI to smallholder farming systems. Moreover, with little AI competence among mycologists, the above barriers further reinforce the significance of these challenges [174]. Filling these gaps is crucial for advancing mushroom research via AI and maximizing its applications in multiple aspects, such as agriculture, pharmaceuticals, and ecology [175].

#### 6.1.4. Limited AI Expertise in Mycology

The significant gap in expertise between the AI community and the mycology community, as well as the mushroom industry, acts as a barrier to the fruitful adoption of AI tools for mushroom research. However, there is a great tragedy in this field where AI has seen significant penetration in other sectors. At the same time, researchers in mycology have minimal exposure to AI and its possible effects [176].

#### 6.1.5. Model Generalization and Interpretability

Recently developed AI models and algorithms experience difficulty in model generalization. Such observation persists when these models are applied to diverse species and fluctuating environmental variables, rendering them incapable of attaining maximum performance. This phenomenon can be attributed to changes in important environmental factors such as light intensity, humidity, and substrate composition, which produce noise and bias in models, thereby lowering prediction accuracy [177]. Additionally, DL architectures operate as uninterpretable “black boxes” retarding the efficiency in model prediction, particularly in biological and agricultural settings [178]. The absence of transparency may lead stakeholders to oppose AI system adoption, particularly in regulatory-sensitive domains such as toxic species detection and food safety grading [179].

#### 6.1.6. Hardware Limitations

On-site deployment of AI systems becomes more complex when deployed to remote and low-resource farming areas [158]. The training and operation of large-scale AI models require high-performance computing (HPC) infrastructure, which proves impractical for mushroom farms in remote areas due to several factors, including high costs, power usage requirements, and maintenance needs [180]. Edge computing represents a promising solution as it can perform computations on-site through embedded devices; however, the current edge AI hardware faces restrictions in memory capacity and processing capabilities [176]. The current hardware limitations prevent the deployment of autonomous decision-making systems that operate in real-time for environmental control, disease surveillance, and growth monitoring tasks. Furthermore, remote farming regions often experience connectivity problems, which prevent the integration of cloud-based AI services, thus requiring innovative solutions that strike a balance between model complexity, energy efficiency, and computational feasibility [181,182].

### 6.2. Future Perspectives

**Figure 7:**
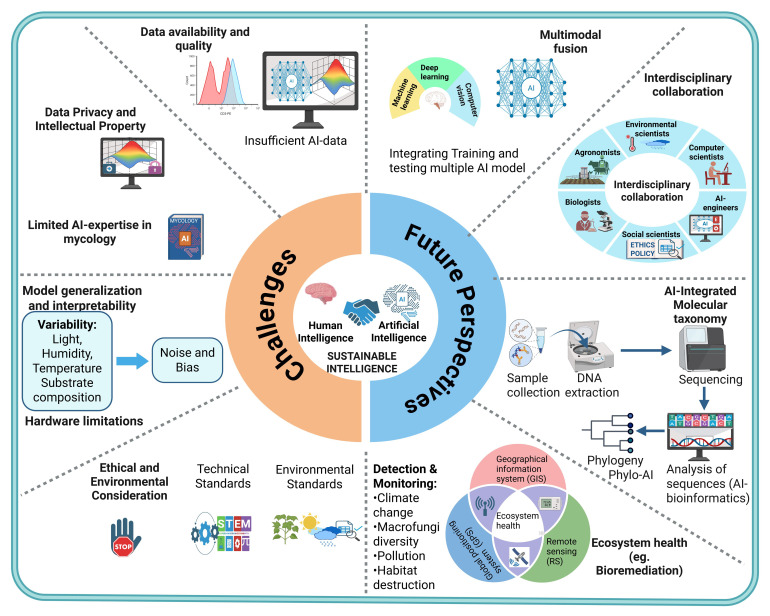
A schematic representation of challenges and potential avenues for sustainable integration of AI in the edible mushroom production cycle.

Innovative research in mushroom science holds great potential for AI applications that scientists have not yet discovered. Advancements in AI technology will uncover new fields that will improve both commercial and ecological significance of mushrooms. Hereunder, we unveil some unquestionably novel areas where AI can outperform in mushroom research, as schematically shown in Figure 7.

#### 6.2.1. Centralized, Open-Access Repositories

A major solution to data availability and accessibility lies in the establishment of centralized, open-access repositories that integrate genomic, phenotypic, cultivation, and environmental data from diverse mushroom species [183]. Collaborative projects among universities, research institutes, and industry can standardize data collection methods, ensuring consistency and reliability [184]. Moreover, applying data augmentation and synthetic data generation techniques can help compensate for limited datasets [185].

#### 6.2.2. Ecosystem Health (Integrating AI with Remote Sensing (RS), Geographical Information System (GIS), and Global Positioning System (GPS) in Predicting and Monitoring Edible Mushroom Ecosystem)

According to Pandey & Pandey [186], a sustainable and healthy mushroom ecosystem depends on the readiness of mycologists to integrate the use of AI technologies, such as drones and remote sensors that are capable of assessing the sustainability of wild mushroom ecology. The combination of smart digital technologies, such as GIS and RS, enhances the practical examination of various ecosystem parameters, including temperature, humidity, contaminants, and invasive species that threaten mushroom survival [187,188]. The evaluation of wild mushroom ecosystem health becomes more accessible by integrating AI with drone and satellite remote sensing technology [189]. The combination of AI with remote sensor data, GIS, and GPS enables the evaluation of soil moisture and temperature, as well as the detection of contaminants and invasive species that affect mushrooms [187,190]. Such developments enable ecologists and conservationists to monitor changes in mushroom biodiversity while predicting the impact of environmental threats such as habitat destruction and climate change on biodiversity [190]. The natural ability of mushrooms to decompose organic matter through bioremediation accelerates development of sustainable waste recycling systems. AI can be harnessed to design and optimize systems that utilize mushroom mycelia for large-scale waste management, including the biodegradation of plastics and organic waste [191]. Harnessing AI technology in monitoring and optimizing these biodegradation processes paves the way for innovative solutions in environmental sanitation, creating new industries based on sustained waste recycling using macro-fungi [192,193].

#### 6.2.3. Utilizing Blockchain and Encrypted Cloud Storage

Data security and privacy depend on secure data-sharing platforms equipped with advanced security features such as blockchain and encrypted cloud storage capable of protecting sensitive datasets [194]. Additionally, developing legal frameworks that recognize and balance the rights of indigenous knowledge holders, researchers, and companies will ensure equitable IP management [195]. Licensing agreements can encourage innovation while safeguarding contributors’ ownership [196,197].

#### 6.2.4. Interdisciplinary Collaboration

AI applications in edible fungi research and production attain their maximum potential when biologists, mycologists, computer scientists, agronomists, and engineers collaborate [195]. Biologists provide domain-specific knowledge essential for interpreting fungal traits, designing experiments, and validating AI outputs. On the other hand, computer engineers demonstrate expertise in algorithm creation, data processing, and model optimization [198]. These collaborative efforts produce AI tools that are both technically sound and biologically meaningful [199].

The interdisciplinary combination of knowledge has already produced successful results. Joint research work has aided in the development of genotype–phenotype prediction systems by merging DL architectures with omics data [200]. The collaboration between experts has resulted in the development of smart cultivation systems by integrating environmental sensor feedback and AI-controlled units to replicate expert decisions [201]. Future initiatives need to establish shared data repositories, open-source platforms, and cooperative training programs to bridge disciplinary gaps. By using XAI techniques, AI models will become more accurate and transparent, gaining the trust of breeders, farmers, and policymakers.

#### 6.2.5. Multimodal Fusion

The effective application of AI for edible fungi research depends on combining multiple data types (multimodal fusion) [202]. Multimodal AI systems use a unified analytical framework to process a combination of heterogeneous datasets, which include genomic profiles, phenotypic images, environmental sensor data, as well as chemical spectra [203]. The complete system optimizes the modeling of complex biological processes that control mushroom growth and adaptation [204]. By integrating environmental sensor data with time-series imaging and genomic data, predictive models can effectively track the growth of mushrooms and provide genotype-based cultivation recommendations [95]. These “holographic” models enable a better understanding of GxE interactions, which results in precision agriculture operations at both species and strain levels [205]. The development of attention-based neural networks and graph neural networks (GNNs) holds promise for effectively handling diverse data relationships [206].

#### 6.2.6. Light Weight AI for Edge Applications

The future of farm deployments depends on researchers’ capacity to develop lightweight AI models capable of solving the deployment challenges. The designed models operate on low-power devices, including Raspberry Pi, Arduino-based systems, and dedicated AI chips such as Google’s Edge TPU [207]. The creation of TinyML confirmed that deep neural networks can be applied to operate directly on microcontrollers while preserving their prediction accuracy [208,209]. The application of lightweight AI models in mushroom farming allows on-site systems to perform autonomous environmental parameter management, disease detection, and mushroom sorting operations independently from cloud connectivity. Researchers use pruning methods, knowledge distillation, and quantization training to achieve AI model compression, which maintains predictive accuracy [210]. As TinyML technology advances, its utility in smart agriculture will continually expand in remote farming areas [211,212].

#### 6.2.7. Ethical and Regulatory Framework for AI-Based Innovations

The development and training of AI should never be carried out to replace human intelligence; instead, it should be used to supplement it, creating a space for collaboration rather than rivalry between the two (Figure 7). The use of AI in various sectors calls for strengthening regulatory frameworks that are robust and sustainable in terms of their ethical application so that human oversight and decision-making are guaranteed [213]. These frameworks must reflect dynamism and adaptability, enabling them to quickly change in response to technological advancements, while simultaneously promoting transparent, accountable, and fair standards of development [214]. Such regulations would strike a balance between the two sides of AI autonomy and human control. Moreover, they would easily pave the way for the harmonious coexistence of AI innovations and human ability toward developing a society that can effectively bring such complements together [215].

#### 6.2.8. AI-Assisted Molecular Taxonomy

Molecular taxonomy represents an exciting future direction for AI research in mushroom science, as it enables the discovery of new mushrooms through advanced gene sequencing and phylogenetic analysis [124,216]. AI surpasses traditional morphological and image-based processing methods by rapidly analyzing extensive genomic data to detect genetic markers while performing advanced phylogenetic analyses with high precision [129]. The graphical model in Figure 7 demonstrates a sustainable AI system that detects minor genetic differences, which enables scientists to discover new species while expanding their knowledge of fungal diversity. AI enables the genetic classification of species through similarity analysis, resulting in efficient and accurate species identification [217]. The method accelerates the discovery of new species while enabling scientists to investigate their ecological roles, potential medical applications, and biotechnological potential [218].

## 7. Conclusions

AI applications in edible mushroom cultivation, breeding and classification mark a revolutionary transformation in fungal research. AI technologies, including ML, DL, and CV, improve yield, disease management, environmental optimization, genetic analysis, species classification, and post-harvest management. In cultivation, AI-based intelligent systems have enhanced efficient environmental control and immediate growth monitoring in mushroom farms. CNNs, IoT, TL techniques, spectral imaging and ML algorithms enhance species identification and automatic quality grading of edible mushroom species. In breeding optimization, an integrated system of AI tools enables researchers to discover suitable genotypes through genomic and phenotypic data analysis, genome editing, and gene mining, thereby optimizing breeding programs.

Nevertheless, several challenges exist in the mushroom value chain, including limited access to well-annotated public datasets, model generalization constraints, and deployment challenges related to hardware. Addressing these barriers requires a multidisciplinary collaboration across different fields of study. Future technological progress is envisioned to emerge from the development of multimodal data fusion methods, lightweight AI solutions for edge computing, and enhanced collaboration between environmental scientists, biologists, and AI engineers. By converging smart agricultural technologies with interdisciplinary research investments, the edible mushroom industry will be propelled toward sustainability, safety, and increased productivity.

## Data Availability

No new data were created or analyzed in this study. Data sharing is not applicable to this article.

## Abbreviations

The following abbreviations are used in this manuscript:

AI	Artificial Intelligence
ML	Machine Learning
DL	Deep Learning
ANN	Artificial Neural Network
CNN	Convolutional Neural Network
DNN	Deep Neural Network
DT	Decision Tree
CV	Computer Vision
MLP	Multi-Layer Perceptrons
RF	Random Forest
TL	Transfer Learning
YOLO	You Only Look Once
k-NN	k-Nearest Neighbor
SVM	Support Vector Machine
ResNet	Residual Neural Network
LSTM	Long Short Tem Memory
AdaBoost	Adaptive Boosting
IoT	Internet of Things
IP	Intellectual Property
PCA	Principal Component Analysis
RL	Reinforcement Learning
XGBoost	A form of Gradient Boost
GxE	Genotype–Environment Interaction
GIS	Geographical Information System
GPS Grad-CAM	Global Positioning System Gradient Weighted Class Activation Mapping
RS	Resmote Sensing
MSI	Multispectral imaging
HPC	High Performance Computing
HI	Hyperspectral Imaging
NIS	Near-Infrared Spectroscopy
RFE	Recursive Feature Elimination
SSR	Simple Sequence Repeats
SNP	Single Nucleotide Polymorphism
SND	Site Directed Nucleases
ZFN	Zinc Finger Nucleases
TALEN	Transcription Activator-Like Effector Nuclease
LLM	Large Language Models
TinyML	Tiny Machine Learning
CRISPR	Clustered Regularly Interspaced Short Palindromic Repeats
GAN	Generative Adversarial Network
t-SNE	t-Distributed Stochastic Neighbor Embedded
NLP	Natural Language Processing
GPU	Graphic Processing Unit
GNN	Graph Neural Network
TPU	Tensor Processing Unit
GPT	Generative Pre-Trained Transformer
UAV	Unmanned Aerial Vehicle
VGG16	Visual Geometry Group 16
RGB	Red, Green, Blue
XAI	Explainable AI
